# Diagnostic Yield and Safety of Bronchoscopic Lung Cryobiopsy in Evaluation of Lung Mass

**DOI:** 10.7759/cureus.19940

**Published:** 2021-11-27

**Authors:** Shafin Babu PS, Vikas Marwah, CDS Katoch, Yadvir Garg, T Ajai Kumar, Manish Sharma, Robin Choudhary, Deepu K Peter, Manu Chopra, Gaurav Bhati

**Affiliations:** 1 Respiratory Medicine, Army Institute Of Cardio Thoracic Sciences, Pune, IND; 2 Respiratory Medicine, Army Institute of Cardio Thoracic Sciences, Pune, IND; 3 Medicine and Respiratory Medicine, Army Institute Of Cardio Thoracic Sciences, Pune, IND; 4 Pathology, Army Institute Of Cardio Thoracic Sciences, Pune, IND; 5 Respiratory Medicine, Command Hospital, Kolkata, IND; 6 Pulmonary Medicine, Army Institute Of Cardio Thoracic Sciences, Pune, IND

**Keywords:** bronchoscopic lung cryobiopsy (blc), advantages of blc, complications of blc, diagnostic yield and safety, mass lesion lung

## Abstract

Background

A mass lesion in the lung is a common finding seen on chest radiology. The prognosis of patients with mass lesions in the lung is capricious as malignancy is a consideration. It is essential to diagnose the underlying aetiology at the earliest with minimally invasive procedures for prompt treatment of the case. Bronchoscopic lung cryobiopsy (BLC) is a newer interventional technique in pulmonary medicine for the diagnosis of mass lesions in the lung.

Materials and methods

This is a retrospective study of patients reporting to a tertiary care centre who were radiologically (by computed tomography scan of the chest) diagnosed with a mass lesion of the lung and who underwent BLC during the period from January 2018 to January 2021. We analysed the diagnostic yield of the technique defined as a positive tissue diagnosis after the histopathological examination (HPE) along with the safety of the procedure.

Results

During the above period, we evaluated 70 patients who were diagnosed radiologically with mass lesions of the lung and underwent BLC. We obtained tissue diagnoses for 66 cases and the result of four cases was inconclusive. The diagnostic yield of the BLC procedure was 94.29%. There was no mortality and complications were minimal bleeding and small pneumothorax.

Conclusion

BLC is a newer technique for obtaining lung tissue via a flexible bronchoscope obviating the need for open lung biopsy. The main advantage of the technique is providing larger tissue samples with minimal or no side effects without undergoing multiple procedures as compared to other bronchoscopic or surgical methods for obtaining a diagnosis from lung tissue. BLC is a safer and promising technique in diagnosing mass lesions of the lung with better yield.

## Introduction

Flexible bronchoscopy was first introduced by Shigeto Ikeda in 1966. After that, bronchoscopy has become an imperative diagnostic and therapeutic device for the management of respiratory diseases. Bronchoscopic lung cryobiopsy (BLC) is a novel video-assisted flexible bronchoscopic technique for obtaining lung biopsy. It comprises the bronchoscopic placement of a flexible cryoprobe into the lung tissue, freezing the cryoprobe and cropping out the lung tissue around the tip under direct vision in a fresh frozen state [1].^ ^Forceps, brushes, or needles are presently the standard devices used during bronchoscopy for taking lung tissue biopsy specimens when diagnosing lung mass [2].^ ^The disadvantage of forceps and needle biopsy is that a relatively small amount of tissue is obtained along with crush artefacts from the instrument tip causing alterations of the tissue samples and affecting the quality of samples for histopathological examination (HPE) [2]. According to the Fleischner Society guidelines, a mass lesion in the lung is defined as a focal pulmonary lesion that is greater than 3 cm in diameter. A mass in the lung is an area of pulmonary opacification that measures greater than 3 cm and is anticipated to be lung carcinoma until confirmed otherwise [3]. There are many causes of a mass lesion in the lung such as lung carcinoma, sarcoidosis, pulmonary tuberculosis, lung carcinoid, and lymphoma. Based on the radiological diagnosis of the lesion, BLC can be done either by endobronchial or transbronchial approach. BLC bears a superficial resemblance to transbronchial lung biopsy (TBLB) or endobronchial lung biopsy (EBLB) using traditional forceps. In BLC, a cryoprobe is introduced through the working channel of the flexible bronchoscope chilled to a very low temperature (about −80° Celsius) for a few seconds and the lung biopsy specimen is taken. The cryobiopsy apparatus works by the principle of the Joule-Thomson effect that freezes a part of the required lung tissue, which remains adherent to the probe and the samples can be taken [4]. BLC is a promising new bronchoscopic lung biopsy technique that helps in obtaining larger and healthier tissue samples from lungs than formerly used traditional forceps biopsy (FB). It is also safer and cost-effective as compared to surgical lung biopsy (SLB). BLC provides tissue specimens larger than the conventional forceps method, which results in a significantly higher diagnostic yield. All patients in our study underwent computed tomography (CT) scan of the chest and were radiologically confirmed cases of mass lesion in the lung and underwent BLC. After obtaining biopsy samples, HPE was performed. The diagnostic yield was defined as obtaining a tissue diagnosis after HPE. According to the definitive clinico-radiological and histopathological evaluation along with multidisciplinary discussion (MDD), the diagnostic yield for BLC was assessed. We also evaluated the complications related to the BLC procedure.

## Materials and methods

**Figure 1:**
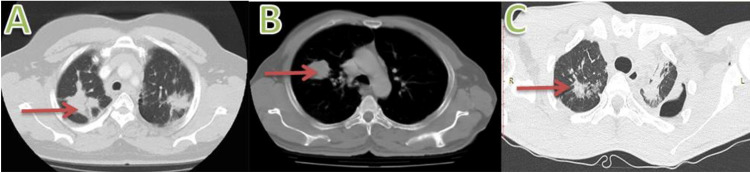
CT scan of the patients. A: Non-small cell carcinoma in the lung. B: Metastatic carcinoma in the lung. C: Endobronchial tuberculosis.

This is a retrospective study of patients reporting to a tertiary care centre during the period from January 2018 to January 2021. Institutional ethical clearance was obtained for the study. During the above specified period, 70 patients diagnosed with a mass lesion in the lung by CT scan of the chest were analysed (Figure 1). The age distribution of the patients is summarised in Table 1 and clinical characteristics are summarised in Table 2.

**Table 1:** Age distribution of the patients.

Age group (years)	Number of patients	Percentage (%)	
≤20	1	1.42	
21-30	4	5.71	
31-40	11	15.70	
41-50	12	17.14	
51-60	20	28.57	
>60	22	31.42	
Total	70	100.00	

**Table 2:** Clinical characteristics of the patients.

S. No.	Predominant clinical characteristics	Number of patients	Percentage (%)
1	Dry cough	20	28.57
2	Cough with expectoration	15	21.41
3	Breathlessness	12	17.14
4	Weight loss	10	14.28
5	Chest pain	08	11.42
6	Lymphadenopathy	05	07.14

There were 68% men as compared to 32% women in the study. The predominant symptoms of patients were cough, breathlessness, weight loss, and chest pain. All patients underwent BLC under conscious sedation. Patients with contraindication for performing BLC were excluded. Contraindications for performing BLC [1] are summarized in Table 3.

**Table 3:** Contraindications of BLC. Absolute contraindications: serial numbers 1 to 7. Relative contraindications: serial numbers 8 to 12. BLC, Bronchoscopic lung cryobiopsy; INR, international normalized ratio.

S. No.	Causes	Parameters
1	Hemodynamic instability	Hypotension or uncontrolled hypertension
2	High‑risk for general anaesthesia (GA) if planned under GA	Category 4 to 6 (American Society of Anesthesiologists)
3	Pulmonary hypertension	Estimated mean pulmonary artery pressure more than 20 mmHg or systolic pressure of >50 mmHg on echocardiography
4	Bleeding diathesis	Platelet count < 50,000 cells/mm^3^ or INR > 1.5
5	Severe hypoxemia in arterial blood analysis	Partial pressure of oxygen (PaO2) in arterial blood less than 50 mmHg on room air
6	Pregnancy	Second and third trimester
7	Diffuse lung disease	With extensive bullae or cysts
8	Anaemia	Haemoglobin less than 8 g/dl
9	Reduced lung function	Forced vital capacity (FVC) less than 1.5 litres or less than 50% predicted
10	Reduced lung function	Forced expiratory volume in 1 second (FEV1) less than 0.8 litres or less than 50% predicted
11	Reduced diffusion capacity of the lung	Diffusing capacity of the lung for carbon monoxide (DLCO) less than 30% predicted
12	Body mass index (BMI)	BMI more than 30 Kg/M^2^

**Figure 2:**
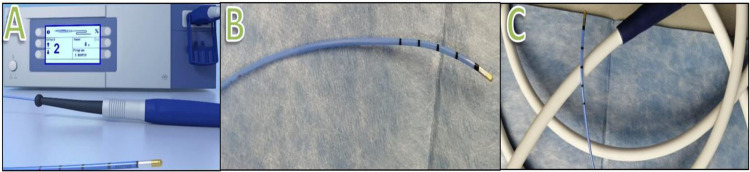
Cryobiopsy tools. A: Cryobiopsy machine with a cryoprobe. B: cryoprobe. C: Cryoprobe with connector.

BLC is a method of obtaining lung tissue samples using a cryoprobe tool (Figure 2), which is usually performed as an outpatient department (OPD) procedure.

**Figure 3:**
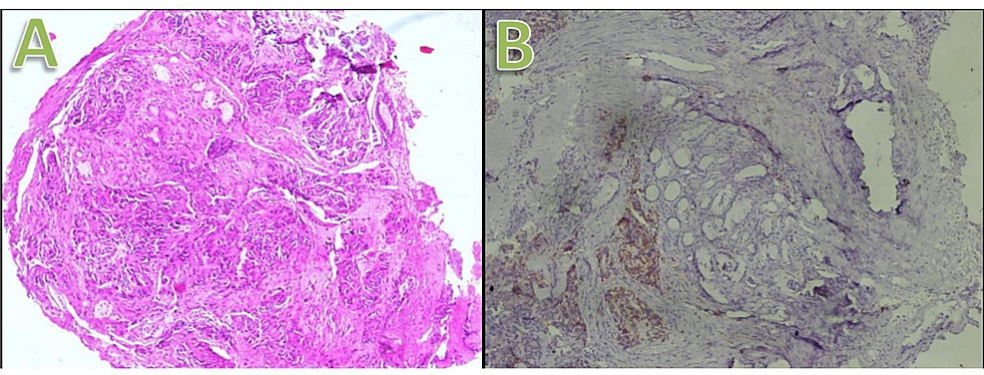
Histopathological diagnosis. A: Non-small cell carcinoma in the lung. B: Primary adenocarcinoma in the lung.

Pre-bronchoscopy protocols were followed. The patient was asked to report fasting for bronchoscopy and written informed consent for the procedure was obtained. Medications influencing the bleeding and clotting diathesis were stopped before intervention. No premedication was administered. Topical anaesthesia included five sprays of 10% lignocaine to the pharynx and 2% of lignocaine gel was instilled into each of the nostrils. Intravenous conscious sedation was administrated with midazolam (maximum 2 mg) and fentanyl (maximum 75 mcg). The patient was pre-oxygenated with 100% oxygen through nasal prong and oxygen was delivered continuously during the procedure. The 2% lignocaine gel was applied over the exterior surface of the bronchoscope to minimize laryngotracheal trauma. Minimal manipulation of the bronchoscope was performed once it was in place. Five millilitre of 2% lignocaine was instilled via transtracheal route through the working channel of the bronchoscope. After bronchoscopic suctioning and clearance of secretions, a flexible 2.4 mm or 1.9 mm cryoprobe was introduced through the working channel of the bronchoscope and advanced into the radiologically diagnosed mass lesion segment of the lung. The probe was retracted by 1-2 cm from the site of the endobronchial lesion and the foot pedal of the cryo station was activated for approximately three to five seconds. This helps the surrounding lung tissue to rapidly freeze and attach to the tip of the cryoprobe. The bronchoscope and cryoprobe with the adherent frozen lung tissue biopsy specimen are then dragged back and removed together from the airway. The cryoprobe is not removed through the working channel of the bronchoscope as doing that may lead to the lung biopsy specimen dropping back into the airway. After this, the assistant separates the biopsy specimen from the cryoprobe and collects it in a formalin fixative. The cryoprobe is then removed from the bronchoscope and then the bronchoscope is reintroduced to check for any active bleeding. After excluding any significant airway bleeding, suction is performed to remove any spilt over blood and the bronchoscope is removed from the airway [5]. An endobronchial balloon blocker was kept prepared in the operation room as it would be accessible to manage bleeding during the lung biopsy. A bedside screening ultrasound of the thorax is performed immediately following the procedure if pneumothorax is suspected. A chest radiograph is obtained later as pneumothorax can occur as delayed complications (preferably after two hours of procedure) [6]. Ensuing biopsies tend to be significantly larger than conventional forceps biopsies and conspicuously lack crush artefacts. Biopsy specimens were fixed in 10% formalin and sent for evaluation by a pathologist and diagnosis was established (Figure 3) [7]. In an uneventful procedure, the patient was discharged home after four hours of the procedure. We evaluated the analytical yield and complications associated with the BLC procedure.

## Results

**Figure 4:**
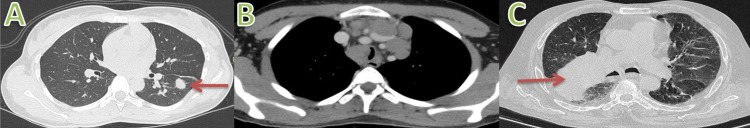
Computed tomography scans of the thorax showing mass lesions in the lung. A: Carcinoid tumour. B: Hodgkin's lymphoma. C: Small cell carcinoma.

We analysed 70 patients who were radiologically diagnosed with a mass lesion in the lung and underwent BLC. Radiologically diagnosed cases with CT scans of the chest are illustrated in Figure 4.

**Figure 5:**
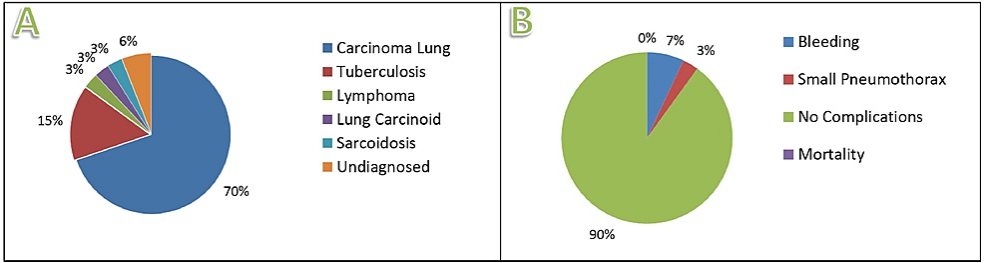
Diagnoses and complications of the cases. A: Diagnoses of the cases. B: Complications of the cases.

Out of the total patients, 66 patients obtained tissue biopsy results and the diagnostic yield of the procedure was 94.28%. Only four patients were undiagnosed, which was 5.72% (approximately 6%) of the total cases. The most common diagnoses of mass lesions in the lung (Figure 5A) were carcinoma (70%), tuberculosis (15%), sarcoidosis (3%), lung carcinoid (3%), and lymphoma (3%). Our study showed a better diagnostic yield as compared to worldwide statistics of conventional bronchoscopic lung biopsies and comparable diagnostic yield with invasive SLB technique. The complication that occurred during the BLC procedure was minimal (Figure 5B). Only two patients had small pneumothorax [8] (size less than 2 cm or 3 cm as per the British Thoracic Society and the American Thoracic Society guidelines, respectively) and five patients had minimal bleeding. The overall complication rate of BLC was 2.8% cases of small pneumothorax and 7.1% of cases with minimal bleeding.

No active intervention was required in any of the post-procedure complications. Bleeding was stopped by conventional method and pneumothorax was resolved by oxygen therapy in all of the cases. We also evaluated the worldwide statistics of impediment rate related to conventional bronchoscopic lung biopsy methods and invasive surgical lung biopsy methods to obtain a tissue diagnosis. Our study showed that the complication rate of BLC as equated to the conventional bronchoscopic method and invasive methods for diagnosis of mass lesions of the lung was lesser or comparable with other techniques. There was no mortality in our study and all patients were discharged to home without any active intervention for complications.

## Discussion

**Figure 6:**
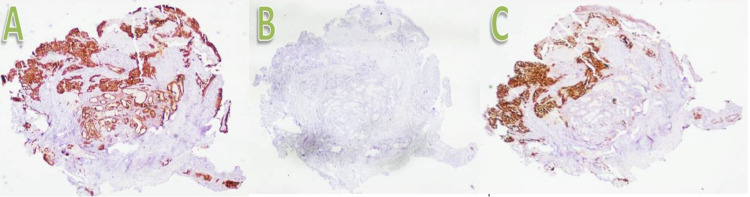
Diagnosis of carcinoma of the lung with tumour markers. A: Cytokeratin 7 positive lung carcinoma. B: Cytokeratin 20 negative lung carcinoma. C: Thyroid transcription factor 1 positive lung carcinoma.

BLC is a promising innovative bronchoscopic lung biopsy technique adept at obtaining bigger and superior preserved samples than previously used conventional methods. Forceps or needle biopsy was previously considered as the customary bronchoscopic approach for diagnosing lung lesions. BLC is a unique technique that helps to obtain larger biopsy samples of lung tissue that surpass the size and standard of forceps biopsy samples with fewer crush artefacts [8]. The cryoprobe was initially devised in surgical applications for cryorecanalization. In the cryoprobe technique, the frozen tissue adherent to the tip of the cryo biopsy probe is mechanically avulsed and allows the abrupt removal of unwanted tissue [9]. BLC employs this same elementary concept that was established in this modern era. There are many reasons for the better quality of BLC compared to other biopsy methods for obtaining lung tissue. First, during the biopsy procedure, the cryoprobe merely needs to gently touch the biopsy site without any squeezing. In FB and SLB, the tissue needs to be penetrated or squeezed, which leads to more mechanical damage. Second, the application of up to −80° Celsius at the cryoprobe’s tip has hemostatic effects in the biopsy specimen, which results in artefact free tissue. Finally, the samples are extracted in a frozen fresh condition without much manipulation. These factors result in bigger and healthier tissue specimens per section and allow accurate evaluation by the pathologist [7]. The diagnosis of carcinoma lung is established and verified with the help of specific tumour markers ​​​(Figure 6).

A mass lesion in the lung is usually diagnosed as lung carcinoma. But there are other entities like tuberculosis, sarcoidosis, carcinoid tumour, and lymphoma, which radiologically mimic lung carcinoma described in other studies. BLC is a prompt and safe procedure in the diagnosis of mass lesions in the lung in conditions radiologically mimicking lung carcinoma [10-12]. BLC could avoid SLBs, which are associated with greater rates of morbidity, mortality, higher cost, and a prolonged hospital stay, which are undesirable to the patient [10]. A comprehensive review of the literature comparing FB and BLC showed that biopsy of the tissue by FB has a diameter of approximately 2 mm and a conclusive diagnosis was achieved in 85.1% of patients. Patients who underwent BLC had a tissue specimen size of more than 7 mm with a diagnostic yield of 95.0% [2]. In one cohort study, the risk of potential procedural complications such as pneumothorax and bleeding was evaluated and found to be more in surgical techniques like open SLB or video-assisted thoracic surgery (VATS) biopsy as compared to BLC [1,13,14]. Another study showed that tissue sampling approaches of BLC appear to be concomitant with a higher analytical yield and a favourable risk-benefit ratio as compared to FB and SLB [15].

The comprehensive and systematic review of the literature suggests that BLC may be considered as a safer and better option than SLB in expert hands [16,17]. Theoretical cost benefits of BLC were analysed in studies in the United Kingdom, Western countries, and America. The data provided by the studies concluded that BLC has the potential to accomplish significant cost savings as compared to SLB or VATS biopsy [18,19].^ ^In exophytic endobronchial lesions, the diagnostic yield and safety of BLC are superior to all other traditional and standard bronchoscopic biopsy methods [20]. Our study holds some limitations. The number of patients was small, but one must bear in mind that patients with mass lesions in the lung are not that frequent. One potential limitation of the BLC procedure is that lesions located in peripheral airways requiring significant flexure of the bronchoscope may not be always reached with the cryoprobe. Our study illustrated that a larger specimen size and better tissue quality along with more diagnostic yield was provided by cryobiopsy. Other advantages of cryobiopsy were cost-effectiveness and safer to the patients as compared to FB and invasive surgical procedures.

## Conclusions

BLC is a novel flexible bronchoscopic technique for obtaining lung biopsy. Our study revealed the efficiency of BLC with a diagnostic yield of 94.28%. The study concluded that bronchoscopic lung cryobiopsy exhibited higher diagnostic yield with minimal complications as compared to other conventional lung biopsy techniques. The main advantage of BLC is providing larger tissue samples with minimal or no side effects without undergoing multiple procedures as compared to other bronchoscopic or surgical methods for obtaining the diagnosis from lung tissue. BLC is a safer, cost-effective, and highly recommended technique in diagnosing mass lesions of the lung with better diagnostic yield as per our study.
